# Comparative Analysis of Human Mesenchymal Stem Cells from Bone Marrow, Adipose Tissue, and Umbilical Cord Blood as Sources of Cell Therapy

**DOI:** 10.3390/ijms140917986

**Published:** 2013-09-03

**Authors:** Hye Jin Jin, Yun Kyung Bae, Miyeon Kim, Soon-Jae Kwon, Hong Bae Jeon, Soo Jin Choi, Seong Who Kim, Yoon Sun Yang, Wonil Oh, Jong Wook Chang

**Affiliations:** 1 Biomedical Research Institute, MEDIPOST Co., Ltd., Seoul 137-874, Korea; E-Mails: genny77@medi-post.co.kr (H.J.J.); byk819@medi-post.co.kr (Y.K.B.); eldjfls3@medi-post.co.kr (M.K.); coiffure@medi-post.co.kr (S.-J.K.); jhb@medi-post.co.kr (H.B.J.); sjchoi@medi-post.co.kr (S.J.C.); ysyang@medi-post.co.kr (Y.S.Y.); wioh@medi-post.co.kr (W.O.); 2 Molecular Biology, University of Ulsan College of Medicine, Seoul 138-736, Korea; E-Mail: swhokim@amc.seoul.kr

**Keywords:** umbilical cord blood, bone marrow, adipo tissue, mesenchymal stem cell, expansion, senescence, anti-inflammation, angiopoietin-1, cell therapy

## Abstract

Various source-derived mesenchymal stem cells (MSCs) have been considered for cell therapeutics in incurable diseases. To characterize MSCs from different sources, we compared human bone marrow (BM), adipose tissue (AT), and umbilical cord blood-derived MSCs (UCB-MSCs) for surface antigen expression, differentiation ability, proliferation capacity, clonality, tolerance for aging, and paracrine activity. Although MSCs from different tissues have similar levels of surface antigen expression, immunosuppressive activity, and differentiation ability, UCB-MSCs had the highest rate of cell proliferation and clonality, and significantly lower expression of p53, p21, and p16, well known markers of senescence. Since paracrine action is the main action of MSCs, we examined the anti-inflammatory activity of each MSC under lipopolysaccharide (LPS)-induced inflammation. Co-culture of UCB-MSCs with LPS-treated rat alveolar macrophage, reduced expression of inflammatory cytokines including interleukin-1α (IL-1α), IL-6, and IL-8 via angiopoietin-1 (Ang-1). Using recombinant Ang-1 as potential soluble paracrine factor or its small interference RNA (siRNA), we found that Ang-1 secretion was responsible for this beneficial effect in part by preventing inflammation. Our results demonstrate that primitive UCB-MSCs have biological advantages in comparison to adult sources, making UCB-MSCs a useful model for clinical applications of cell therapy.

## 1. Introduction

Mesenchymal stem cells (MSCs) possess self-renewal and multi-lineage differentiation potentials, and are thus an attractive source of stem cells for tissue engineering [1,2]. Although adult bone marrow (BM) and adipose tissue (AT) are the main sources for clinical use, they are limited because harvesting requires invasive procedures and there are stringent donor age requirements [3,4]. MSCs derived from elderly patients may be clinically ineffective. Therefore, alternative sources have been strongly pursued in primitive or neonatal tissues, including placenta, umbilical cord, and amnion. However, these tissues require complex processing for cell isolation [5–7].

Umbilical cord blood (UCB) is considered most suitable because it is free from ethical complications, and easy to isolate via non-invasive methods. UCB produces large yields of MSCs and possess immunosuppressive activities, making it useful in allogeneic settings [8,9]. Thus, its application has been attempted in a wide spectrum of diseases. We recently demonstrated the therapeutic efficacy of UCB-MSCs in various disease models [10–14]. UCB-MSCs exhibit characteristics similar to BM-MSCs and AT-MSCs, including fibroblastoid morphology, surface proteins, and differentiation potential as defined by the proposed International Society for Cellular Therapy (ISCT) criteria [15,16]. However, the ISCT criteria lack information of MSCs’ potential as therapeutic cell sources. Therefore, we must evaluate the potential of MSCs from various origins to select the best source for cell-based therapy. Comparative studies of the therapeutic potential of UCB-MSCs and other MSCs are lacking. As the number of clinical trials and variety of adult cells used in regenerative therapy increases, cell therapeutic potential will be a primary concern.

MSCs proliferation and senescence have been considered important issues by scientists seeking to use them for therapeutic purposes. *Ex vivo* expansion of MSCs is required to generate a pure cell population in an amount sufficient for the clinical indication. During this expansion process, cells enter cellular senescence, which leads to a gradual reduction of their potency and significant changes in protein expression [17,18]. However, studies of cellular senescence in various MSCs have not been published. It is also necessary to understand the mechanism by which MSCs induce their physiological effect, as this may explain the key mechanisms of tissue repair. Mechanistic evidence of the benefits of MSCs has been based on their paracrine effect, controlling inflammation or promoting regeneration via soluble factors [19,20]. There is convincing evidence that MSCs from diverse tissue are different. However, the benefits and mechanisms of these variously sourced MSCs remain unexplored.

To the best of our knowledge, this is the first study to directly compare the therapeutic potentials of BM-, AT-, and UCB-MSCs in the context of cellular senescence, anti-inflammatory properties, and secreted proteins.

We examined cell expansion, surface markers, immune suppression, multi-differentiation potential, senescence, anti-inflammatory activity, and secreted factors in UCB-MSCs (*n* = 12), BM-MSCs (*n* = 12), and AT-MSCs (*n* = 7) from many donors under the same conditions. Such information may be crucial to identify the most suitable cell source for a given clinical application.

## 2. Results and Discussion

### 2.1. Characterization of MSCs from BM, AT, and UCB

**Figure 1:**
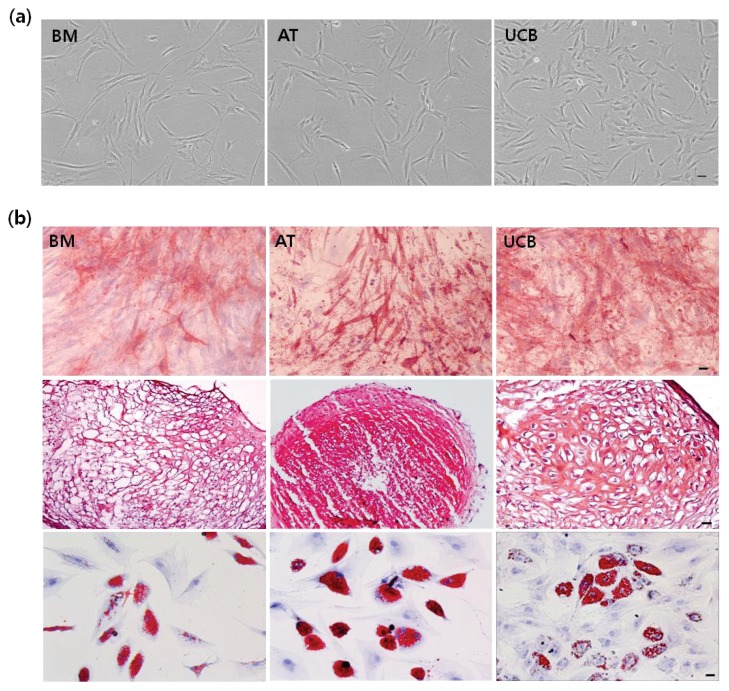
Characterization of mesenchymal stem cells (MSCs) from bone marrow (BM), adipose tissue (AT), and umbilical cord blood (UCB) in Passage 5. (**a**) Morphology of cultured BM-, AT-, and UCB-MSCs. All MSCs exhibited spindle-shaped morphology. Scale bar = 50 μm; and (**b**) During incubation in specialized induction media, multi-lineage differentiation was measured by staining for typical lineage markers. In MSCs from three sources, osteogenic cells were evaluated by bone type alkaline phosphatase staining (top). Chondrogenic cells accumulated sulfated proteoglycan that stained with safranin O (middle). Adipogenic cells accumulated lipid vacuoles within the cytoplasm that stained with oil red O (bottom). Scale bar = 50 μm (Magnificiation: 100×).

To determine whether UCB-MSCs share stem cell properties with BM-MSCs or AT-MSCs, we compared their morphology and surface markers. In Passage 5, UCB-MSCs exhibited a spindle-shaped morphology comparable to that of BM- and AT-MSCs (Figure 1a). FACSCalibur flow cytometer (FACS) analysis revealed all MSCs were positive for expression of cluster of differentiation (CD29), CD44, CD73, CD90, CD105, and human leukocyte antigen (HLA)-ABC, but negative for CD14, CD19, CD34, CD45, CD79a, or HLA-DR, according to ISCT criteria (Table 1). To investigate the differentiation capacity of variously derived MSCs, cells were cultured in osteogenic, adipogenic, and chondrogenic differentiating media. For example, the three-lineage potential for osteogenesis, chondrogenesis, and adipogenesis is a standard for defining multi-potent MSCs. Tri-lineage potential in all MSCs was tested by staining for typical lineage marker. Osteogenesis was defined by a bone-type marker, determined by alkaline phosphatage (ALP) staining; chondrogenesis, characterized by an increase in proteoglycans, was demonstrated by safranin O staining; adipogenesis was observed by staining of cytoplasmic lipid droplets with oil Red O (Figure 1b). These results confirm that MSCs from three sources can successfully differentiate into multiple cell types including osteoblasts, chondrocytes, and adipocytes. Direct comparison of UCB- to BM- and AT-MSCs demonstrated the similar pattern of extent and level of original MSCs features.

### 2.2. Growth Profiling and Cellular Senescence

**Figure 2:**
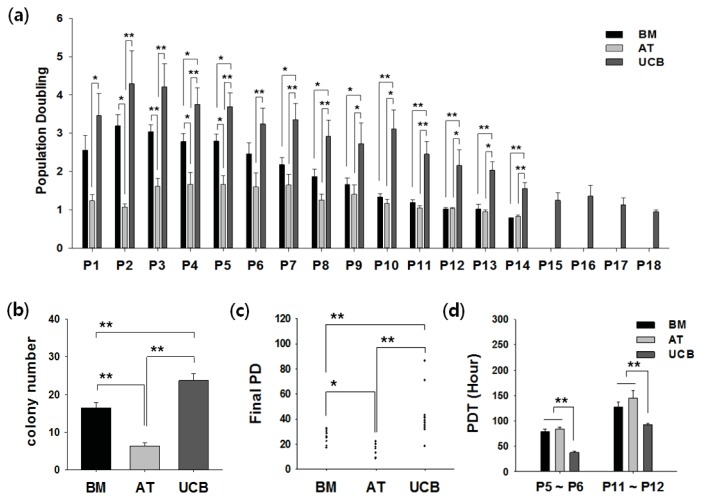
Growth kinetics of BM-, AT-, and UCB-MSCs. (**a**) UCB-MSCs showed more population-doubling (PD) than other tissues in all passages; (**b**) Clonogenetic capacity was measured by colony forming unit-fibroblast (CFU-F) assay. UCB-MSCs formed more colonies than BM- or AT-MSCs in Passage 3; (**c**) Comparison of final PD indicates maximal expansion potential. (**a**–**c**) Error bars represent the means ± SD. BM (*n* = 12), AT (*n* = 7), UCB (*n* = 12); *p < 0.05, **p < 0.01; and (**d**) Each population doubling time (PDT) was analyzed in Passage 5 to 6 and in Passage 11 to 12, respectively. In both passages, the PDT of UCB-MSCs was significantly lower. Error bars represent the means ± SD, *n* = 5; **p < 0.01; P, passage.

For therapeutic purposes, large-scale expansion and slow senescence are important. Here, we determined the cell proliferation rate and cellular senescence in all isolated MSCs. Cells were cultured until growth ceased. Population-doubling (PD) was measured for every passage (Figure 2a). UCB-MSCs could be cultured for significantly longer periods and exhibited the greatest expansion capacity, whereas AT-MSCs had the shortest culture time and lowest growth rate. In most BM- and AT-MSCs, cell growth arrested in Passage 11–12, whereas UCB-MSC proliferation stopped in Passage 14–16. A comparison of the clonogenic ability in different tissues by colony forming unit-fibroblast (CFU-F) assay showed that more colonies formed from UCB-MSCs (23.7 ± 5.8) than from BM-MSCs (16.5 ± 4.4) or AT-MSCs (6.4 ± 1.6) in Passage 3 (Figure 2b). Growth profiling of all MSCs was summarized as the final PD number through long-term cultivation. The final PD of the AT-MSCs was found to be significantly less than that of the BM and UCB. Therefore, UCB-MSC is the highest proliferative MSC among BM-MSC and AT-MSC (Figure 2c). Population doubling time (PDT) was calculated in Passages 6 and 12; at both points, the shortest PDT was found in UCB-MSCs (Figure 2d).

**Figure 3:**
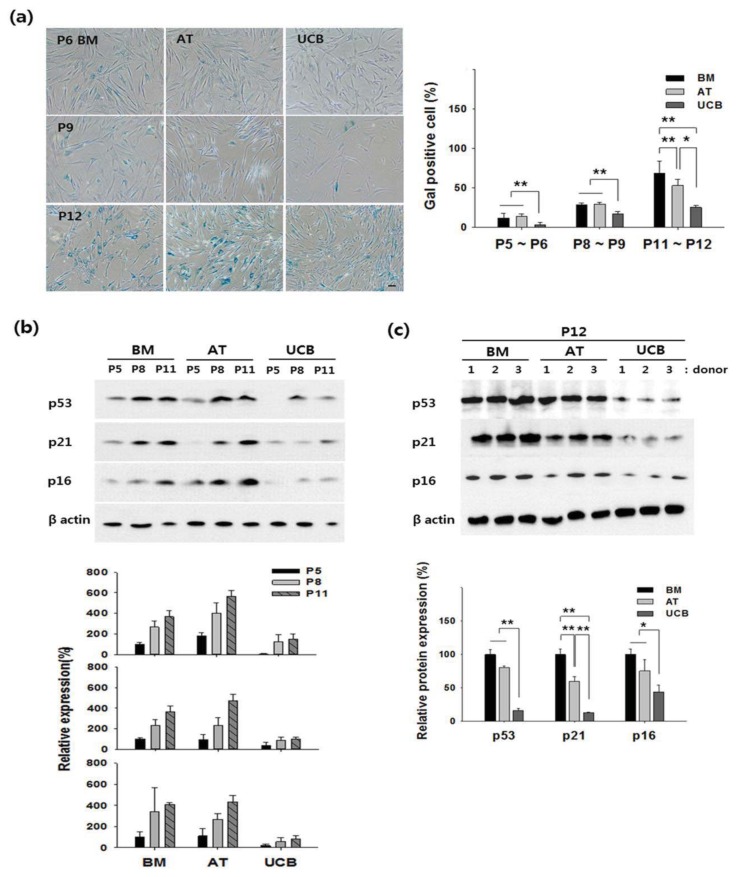
Cellular senescence of BM-, AT-, and UCB-MSCs. (**a**) Senescence stage evidenced by positive Gal staining in P6, P9 and P12. Staining was quantified by positive cell count. Error bars represent the means ± SD, *n* = 5; *p < 0.05, **p < 0.01; (**b**) Expression of senescence-associated proteins (p53, p21, and p16) in each MSC was gradually increased during cell expansion (P5, P8 and P11); and (**c**) In Passage 12, protein expression was measured by Western blotting. (**b**–**c**) β-actin was used as an internal control. Results were quantified by densitometry with signals normalized to the standard signal (BM), set as 100%. UCB-MSCs expressed significantly lower levels of senescence-associated proteins. Error bars represent the means ± SD, *n* = 3; *p < 0.05, **p < 0.01; P, passage.

To determine whether various features of cellular senescence were similar in BM-, AT, and UCB-MSCs, we tested senescence-associated β-galactosidase staining (Gal staining) and senescence-related protein in several donors. First, Gal staining revealed almost no positive cells in UCB-MSCs, while the mean in BM- and AT-MSCs was 11% to 13% by Passage 6. Also, Gal expression dramatically increased in all MSCs from Passages 9 to 12, but the level in UCB-MSCs was significantly lower than in the comparators (Figure 3a). Second, because cell cycle regulators are associated with stem cell senescence, we observed senescence-related proteins p53, p21, and p16 by Western blotting. In three sources of stem cells, expression of p53, p21, and p16 were gradually increased during the indicated passage of culture expansion (Passages 5, 8 and 11). However, expression of these proteins was lower in UCB-MSCs, compared to that of BM- and AT-MSC (Figure 3b). In addition, we confirmed expression level of three proteins in Passage 12 of each MSC from three different donors. BM-MSCs and AT-MSCs strongly expressed p53, p21, and p16, but UCB-MSCs weakly expressed these proteins (Figure 3c). Thus, UCB-MSCs could be maintained for the longest period. Also, UCB-MSCs exhibited the highest PD in all passages analyzed and expanded later than the comparators.

### 2.3. Anti-Inflammation and Angiopoietin-1 Secretion

**Figure 4:**
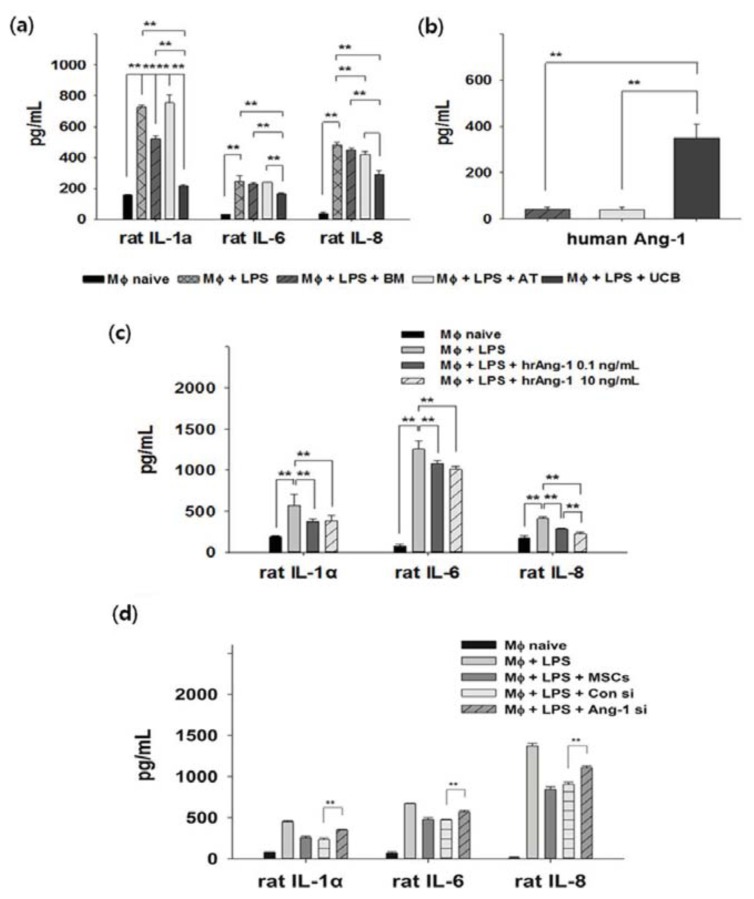
Anti-inflammatory effects of BM-, AT-, and UCB-MSCs in an LPS-induced inflammatory model. (**a**) Rat alveolar macrophages were stimulated with LPS and co-cultured with MSCs. The co-cultures were maintained for 3 days and the supernatants were analyzed for inflammatory cytokines (rat IL-1α, IL-6 and IL-8) by ELISA. UCB-MSCs exhibited significantly lower levels of inflammatory cytokines. Error bars represent means ± SD, *n* = 5; **p < 0.01; (**b**) Ang-1 secretion. UCB-MSCs secreted a significantly higher level of Ang-1; (**c**) Human recombinant Ang-1 (hrAng-1) significantly reduced expression of inflammatory cytokines in LPS-treated macrophages; and (**d**) Pretreatment of siRNA for Ang-1 significantly reduced anti-inflammatory effect of UCB-MSC in a co-culture system; (**b**–**d**) Error bars represent means ± SD, *n* = 3 per group; *p < 0.05, **p < 0.01; MΦ, macrophage.

To quantitatively analyze the paracrine anti-inflammatory properties of MSCs, rat alveolar macrophage NR8383 cells were used as an *in vitro* model. LPS-activated macrophages were co-cultured with MSCs from different sources. Enzyme-linked immunosorbent assay (ELISA) revealed up-regulation of inflammatory cytokines IL-1α, IL-6, and IL-8 in NR8383 after LPS stimulation. Secretion of these cytokines was significantly down-regulated in the UCB-MSCs treatment group (Figure 4a). Next, to investigate whether the observed anti-inflammation effect was connected to secreted proteins, we analyzed the cell culture medium for the presence of secreted proteins with reported anti-inflammatory activity [21,22]. Human interleukin 1 receptor antagonist (IL-1ra), stromal derived factor-1α (SDF-1a), and platelet-derived growth factor (PDGF) were negative or expressed at very low levels in co-culture conditions, insufficient to demonstrate anti-inflammatory potential. At the same time, there was no difference in expression of keratinocyte growth factor (KGF) (data not shown). However, UCB-MSCs stimulated by co-culture with LPS secreted a significantly higher level of angiopoietin-1 (Ang-1, Figure 4b). Since Ang-1 may have an anti-inflammatory role, we analyzed whether human recombinant Ang-1 (hrAng-1) rescues LPS inflammation. LPS-stimulated NR8383 were treated with hrAng-1 (0.1 or 10 ng/mL). Compared to the positive control, secretion of IL-1α, IL-6, and IL-8 were significantly down-regulated in the hrAng-1 treatment group (Figure 4c). In order to confirm the role of Ang-1 in anti-inflammatory effect of UCB-MSCs, small interference RNA (siRNA) of Ang-1 was pre-treated in UCB-MSCs and these were co-cultured with LPS-activated macrophage. Inhibitory effect for secretion of Ang-1 by siRNA was confirmed by ELISA (Figure S1). To check induction of inflammatory condition in LPS-activated macrophage, released inflammatory cytokines such as IL-1α, IL-6, and IL-8 in conditioned media were measured by each specific ELISA. Although control siRNA treated UCB-MSCs or naïve UCB-MSCs reduced the level of three inflammatory cytokines in a co-culture system, these effects were reversed by siRNA of Ang-1. These results strongly indicate differences in the anti-inflammatory potential of MSCs from different sources, and demonstrate the superior capacity of UCB-MSCs. Furthermore, our data suggest Ang-1 secreted by UCB-MSCs provides an anti-inflammatory effect in LPS-stimulated cells.

### 2.4. Discussion

Over the last few years, BM, AT, and UCB have become readily accessible sources of cell-based therapy for utilization in tissue engineering. Multiple efforts by different laboratories have aimed to identify and compare the cellular and molecular aspects of cell morphology, surface markers, and differentiation capacity of stem cells from these various sources [16,23]. Kern *et al.* [23] concluded that AT is suitable as it contains MSCs at the highest frequency and UCB as it seems to be expandable to higher numbers. However, these studies seem to be focused on an understanding of biology of various MSCs. A major concern that remains is to determine which cell source is most appropriate and effective. In our report, we compared UCB-MSCs to MSCs from BM and AT, which exhibit typical MSCs characteristics. All cells exhibited the original MSCs features as defined by the ISCT minimum criteria: spindle shape, multi-lineage differentiation, and surface marker expression [15]; Here, we also directly compare the therapeutic potentials of BM-, AT-, and UCB-MSCs in the context of cellular senescence, anti-inflammatory properties, homing effect to injury site and paracrine action.

The growth kinetics and clonality of UCB-MSCs was significantly higher and these cells could be propagated for longer periods in culture than comparator MSCs (Figure 2). Greater proliferation of MSCs allowing for shorter culture time or large expansion to generate a sufficient population of MSCs for clinical use would clearly be beneficial. In fact, BM and AT are reliable sources for expanding MSCs in autologous settings, as all preparations gave rise to MSCs. By definition, autologous cell therapy has been limited to these adult stem cells. In particular, Zhang *et al.* reported that UCB-MSCs obtained from a single donor were sufficient to generate 1 × 10^9^ cells within Passage 3–4 in a cell factory system [24]. For clinical application, cell doses of 1 × 10^9^ cells are desirable, making UCB-MSCs a useful model in allogeneic settings. In addition, UCB-MSCs are able to expand over 16 passages with normal karyotype [24,25]. Taken together, the higher growth rate of UCB is thought to reflect the relatively primitive source of MSCs compared to adult BM and AT.

After extensive culture expansion, senescence becomes important; it is defined by growth arrest and differentiation loss of MSCs. For the first time, we demonstrated that senescence of UCB-MSCs was slower than that of BM- or AT-MSCs, indicating the superiority of UCB-MSCs. Gal staining revealed significantly less of this senescence marker in UCB-MSCs than in the comparators (Figure 3a). In addition, senescence can be trigged by p53 and its downstream target p21, followed by activation of p16 [26,27]. In our study, expression of p53, p21, and p16 was measured in Passage 12. According to the literature, higher passages, namely p12, are considered “late” passage [28]. These proteins were strongly expressed in BM- and AT-MSCs, consistent with previous reports. However, expression of these proteins was significantly lower in UCB-MSCs (Figure 3b). In Passages 14–16, UCB-MSCs exhibited strong expression of p53 and p21 and weak expression of p16 (data not shown). This may suggest senescence of UCB-MSCs is not correlated with p16. In some BM-MSCs, low p16 expression has been demonstrated during long-term culture [29]. It has also been suggested that senescence patterns differ between MSCs sources [30]. We also observed that senescence was age-dependent, as described by Zaim *et al.* [31]. Many authors have suggested that MSCs from neonatal tissues showed no sign of cellular senescence over long-term culture [32]. Neonatal tissues exhibit certain biological properties that differ from MSCs originating from adult sources. Several studies have found remarkable decrease in stem cell characteristics with increasing donor age in humans MSCs [17]. Further study of senescence in MSCs may help address these issues.

Finally, it is essential to understand the mechanism of MSCs-induced physiological effects, since this may explain the several mechanisms of tissue repair. Most early studies asserted that the mechanical effect was from the capacity of MSCs to migrate to damaged tissues and differentiate into specific cell lineages such as bone or cartilage [33]. In our data, MSCs from three sources have a strong migratory ability toward site of inflammation (Figure S2). Lately, the mechanistic role underlying the therapeutic benefit of MSCs changed principally to the paracrine role of secreted factors [34]. Inflammatory disease is a suitable model for comparison of paracrine effects; thus, we used a macrophage model with LPS stimulation. Several studies have suggested MSCs of different origins inhibit LPS-induced inflammation *in vivo* and *in vitro* [35,36]. Our study therefore demonstrated that MSCs derived from BM, AT, and UCB have significantly different anti-inflammatory capacities and confirmed that UCB-MSCs exhibit the greatest anti-inflammatory effect (Figure 4a). Another study supported this finding in a spinal cord injury model [37]. Moreover, we also searched for the main anti-inflammatory secreted factor based on the other report which suggests MSCs secrete various soluble factors during inflammation [22]. Ang-1, one of the major inflammatory factors, controls important anti-inflammatory processes such as *in vitro* and *in vivo* experimental lung injury [38,39]. Our study revealed that it was also secreted most highly by UCB-MSCs in a pathological model (Figure 4b). To verify the anti-inflammation role of Ang-1, we investigated whether recombinant Ang-1 reduces LPS inflammation. We obtained data that treatment with recombinant Ang-1 inhibited LPS inflammation (Figure 4c). Moreover, blocking of Ang-1 secretion by siRNA reduced the anti-inflammatory effect of UCB-MSC in inflammatory condition (Figure 4d). Intriguingly, secretion of Ang-1 was closely associated with up-regulation of anti-inflammatory factors, which ultimately affect the outcome of the paracrine effect by UCB-MSCs, suggesting UCB contains more anti-inflammatory effect than BM or AT. In addition, MSCs have immune-regulation effects through the production of several soluble factors [40]. In our comparative study, the high immune-suppression effect of UCB-MSCs was similar to the comparators by mixed lymphocyte reaction (MLR, Figure S3). Therefore, these results indicated that UCB-MSCs may be useful as an alternative source in the treatment of inflammatory disease such as acute lung injury (ALI) or acute respiratory distress syndrome (ARDS).

Despite the many merits of UCB-MSCs, their utility remains controversial because of the low isolation efficiency. Many groups have reported that UCB has a maximum isolation efficiency of 65% in various culture methods; depletion of lymphocytes and monocyte from mononuclear cell (MNC) before cell seeding, addition of cytokines supplements or platelet lysate to the medium, or cultivation of cells under hypoxia [25,41–44]. However, the success rate can be increased above 90% by using density gradient purification [24]. This could also help improve the utility of UCB-MSCs as a therapeutic resource. Further studies should be performed to validate these methods for clinical utilization.

## 3. Experimental Section

### 3.1. Cell Culture

This study was approved by the Institutional Review Board of MEDIPOST Co., Ltd. (Seoul, Korea).

BM-MSCs: Bone marrow (BM) was isolated by aspiration from 10 donors aged 20 to 30 years old, with informed consent. BM aspirates were isolated over Ficoll-Hypaque solution (*d* = 1.077 g/cm^3^; Sigma, St. Louis, MO, USA) [23].

AT-MSCs: Adipose tissue (AT) from 5 donors, aged 23 to 40 years, was obtained during elective liposuction procedures with informed consent. AT was isolated using collagenase (type I, Sigma) [23].

UCB-MSCs: Umbilical cord blood (UCB) was collected from umbilical veins after neonatal delivery with maternal informed consent (*n* = 24). All UCB were collected within 24 h. UCB was isolated by separating mononuclear cells (MNCs) with Ficoll-Hypaque solution [23].

Mononuclear cells were isolated by centrifugation in a Ficoll-Hypaque gradient (*d* = 1.077 g/cm^3^; Sigma). The separated mononuclear cells were washed, suspended in a-minimum essential medium (a-MEM, Gibco, Carlsbad, CA, USA), supplemented with 10% fetal bovine serum (FBS) (Gibco), and seeded at a concentration of 5 × 10^5^ cells/cm^2^. Cultures were maintained at 37 °C in a humidified atmosphere containing 5% CO_2_ with a change of culture medium twice a week. After cultivation, both isolating frequency for BM and AT were found be 100%, while that of UCB were 50%.

Primary BM-MSC or AT-MSC controls: BM-MSCs (2 lots) were purchased from Cambrex (Walkerville, MD, USA). AT-MSCs (2 lots) were purchased from American type culture collection (ATCC, Rockville, MD, USA) and Promocell (Heidelberg, Germany).

In time-dependant cell cultivation, the expansion of cell was analyzed by using the trypan blue exclusion method. In each passage, MSC was cultured for 7 day; cells were collected by trysin-EDTA (Gibco), counted and reseeded with the initial cell density (2000 cells/cm^2^). Culture medium was replaced twice weekly. The number of PD (population doubling) was calculated based on the total cell number at each passage; PD was calculated for every passage by dividing the logarithm of the fold increase value obtained at the end of the passage by the logarithm of 2. This procedure was repeated until the cell stopped proliferating. At this point, number of cells was counted to calculate the final PD [29].

Population doubling time (PDT) was examined using the formula: (*t* – *t*_0_)·log2/log(*N* – *N*_0_), where *t* – *t*_0_ is culture time (h), *N* the number of harvested cells and *N*_0_ is the number of cells in the initial.

CFU-F (colony forming unit-fibroblast) assay was performed by seeding cells in a culture dish (BD Biosciences, San Jose, CA, USA) and incubating in humidified 5% CO_2_ at 37 °C; culture medium was exchanged every 3 days. After 2 weeks, the dishes were washed twice with phosphate-buffered saline (PBS, Invitrogen, La Jolla, CA, USA), fixed with 100% methanol, and stained with 3% Crystal violet (Sigma). The number of colonies was counted.

### 3.2. Immunophenotyping

For cytometric analysis of cultured cell phenotypes, cells were stained with antibodies against human CD14, CD19, CD34, CD45, HLA-DR (FITC, BD Biosciences), CD29, CD44, CD73, CD90, CD146, CD271 (PE, Pharmingen, Los Angeles, CA, USA), and CD105 (PE, Serotec, Kidlington, UK) for 15 min at room temperature. Corresponding mouse isotype antibodies were used as controls. The cells were washed with PBS and fixed with 1% (*v*/*v*) paraformaldehyde (PFA). MSCs immunotypes were determined by FACS instrument (BD Biosciences) and the percentage of expressed cell surface antigen was calculated for 10,000 gated-cell events.

### 3.3. *In Vitro* Multi-Lineage Differentiation

Multi-lineage potential was assessed by incubating cells under specific conditions to induce differentiation into osteoblasts, chondrocytes, and adipocytes [45]. After differentiation, multi-lineage potential was evaluated [45]. Briefly, osteoblast formation was assessed by measuring the level of alkaline phosphatase (ALP) staining (Sigma); chondrocyte formation was determined by safranin O staining (Sigma); adipocyte formation was assessed based on staining of accumulated lipid vacuoles with oil Red O (Sigma).

### 3.4. Senescence-Associated β-Gal Staining (Gal Staining)

Senescence-associated β-galactosidase staining (Gal staining) was used as a biomarker of senescence in MSCs. Gal activity was qualitatively assessed with a histochemical staining kit (Sigma) per the manufacturer’s instructions, followed by inversion microscopy. The percentage of senescent cells was represented by the number of stained cells in the total population.

### 3.5. Western Blotting

Cell extracts were prepared in buffer containing 9.8 M urea, 4% CHAPS, 130 mM dithiothreitol, 40 mM Tris-HCl, and 0.1% sodium dodecyl sulfate (SDS). Protein concentration was measured by bicinchoninate assay (BCA, Sigma). Protein extract (10 μg) was separated by sodium dodecyl sulfate-polyacrylamide gel electrophoresis (SDS-PAGE, Invitrogen) and the resolved proteins were transferred to nitrocellulose membranes. Each membrane was incubated with anti-Phospho53 (p53, Cell signaling, Danvers, MA, USA), anti-p21 (Cell Signaling), anti-p16 (Cell Signaling), and anti-β actin (Sigma).

### 3.6. Inflammatory Condition *in Vitro*

The rat alveolar macrophage cell line NR8383 (ATCC) was cultured in Roswell Park Memorial Institute (RPMI) supplemented with 10% fetal bovine serum (FBS, Invitrogen). Cells were maintained at 37 °C in a humidified atmosphere of 5% CO_2_. Lipopolysaccharide (LPS) (1 μg/mL; Sigma) was used to activate NR8383, serving as a positive control for inflammation. LPS-exposed NR8383 were co-cultured with MSCs or human recombinant angiopoitin-1 (hrAng-1, R&D Systems, Minneapolis, MN, USA) for 72 h. Cytokines was measured in the co-culture supernatants. Rat interleukin-1α (IL-1α), rat IL-6, rat IL-8, and human angiopoietin-1 (Ang-1) were quantified by Enzyme-linked immunosorbent assay (ELISA, R&D Systems) according to manufacturer protocols. Results were acquired by measuring absorbance at 450 nm.

### 3.7. Small Interfering RNAs (siRNA) Treatment

For siRNA experiments, Ang-1 or control siRNA were designed by Dharmacon (Chicago, IL, USA). siRNA was treated with the Dharmafect reagent (Dharmacon) according to the manufacturer’s protocol.

### 3.8. Statistical Analyses

All data are reported as mean ± standard deviation (SD) and were analyzed by SPSS (version 18, SPSS Inc., Chicago, IL, USA). Differences and significance were verified by one-way ANOVA followed by the Fisher’s least significant difference (LSD) *post hoc* test. *p*-values less than 0.05 were considered statistically significant.

## 4. Conclusions

In summary, we suggest MSCs from various sources have different therapeutic potential. UCB-MSCs have a longer culture, a large scale expansion, a retardation of senescence, and a higher anti-inflammation effect via Ang-1 than other MSCs. Therefore, our results demonstrate that primitive UCB-MSCs have biological advantages in comparison to adult sources, making UCB-MSCs a useful model for clinical applications of cell therapy.

## Supplementary Information



## Figures and Tables

**Table 1:** Surface markers are indicated as positive or negative in the box.

Marker	CD11b	CD14	CD19a	CD34	CD45	CD79a	HLA DR	CD29	CD44	CD73	CD90	CD105	CD166	HLA ABC
BM	−	−	−	−	−	−	−	+	+	+	+	+	+	+
AT	−	−	−	−	−	−	−	+	+	+	+	+	+	+
UCB	−	−	−	−	−	−	−	+	+	+	+	+	+	+

+: more than 95%; −: less than 5%.
