# Supplementary material for: Comparative Analysis of Human Mesenchymal Stem Cells from Bone Marrow, Adipose Tissue, and Umbilical Cord Blood as Sources of Cell Therapy
Source: Int J Mol Sci. 2013 Sep 3;14(9):17986–8001. doi: 10.3390/ijms140917986 (PMC3794764; doi:10.3390/ijms140917986)
Supplement: Supplementary file 1 [file ijms-14-17986-s001.pdf]

Supplementary Information

Figure S1. Inhibition of Ang-1 secretion by siRNA in UCB-MSCs. Reduced secretion of Ang-1 was measured by ELISA. Error bars represent the means ± SD, $n = 3$; ** $p < 0.01$ *vs*. control siRNA.

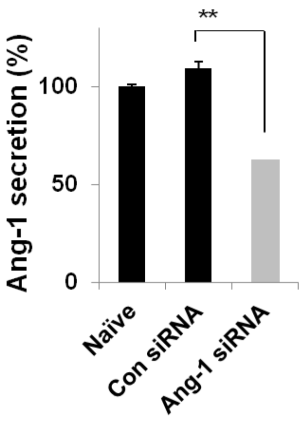

Migration Assays via a Transwell System

MSCs from three sources (BM, AT, UCB) were labeled with PKH26 (Sigma). Labeling was performed according to recommended protocol. In brief, cells were suspended in Diluent C at a density of 2×10^6 cells/mL and mixed with an equal volume of the PKH26 dye in Diluent C. Labeling was carried out at room temperature for 4 min. The labeling reaction was terminated by the addition of an equal volume of FBS. For inflammatory condition *in vitro*, we used LPS-exposed NR8383, as described in Expression section. MSCs migration was analyzed using transwell migration assays (FALCON). This permitted separation of the two plate chambers with a porous membrane (8.0 μm size) was cultured. PKH26-labeled MSCs (1×10^4 in 500 μL medium) were added to the upper chamber with NR8383 or LPS-exposed NR83883 (lower chamber). The chambers were incubated at 37 ℃ for 72 h. Then, the numbers of MSCs that had migrated into the lower chamber were counted.

Figure S2. Migration assay of MSCs from BM, AT, and UCB. (**a**) Analysis of ELISA revealed up-regulation of inflammatory cytokines IL-1α, IL-6, and IL-8 in NR8383 after LPS stimulation. Error bars represent the means ± SD, $n = 3$; ** $p < 0.01$ *vs*. NR8383; and (**b**) MSCs from three sources and LPS exposed NR8383 were co-cultured in the upper and lower chambers respectively. After 72 h, migrated MSCs in a lower chamber were counted under the microscope.

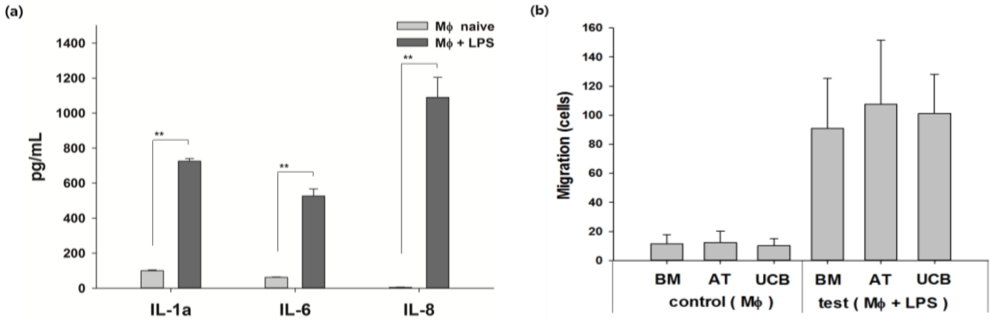

Mixed Lymphocyte Reaction Assay (MLR)

To assess T-cell reactivity against allogeneic cell populations, human responder peripheral blood mononuclear cells (PBMCs) were co-cultured with inactivated allogeneic PBMCs or BM-, AT-, or UCB-MSCs in 96-well plates. PBMCs were purchased from Astarte Biologics, LLC (Bellevue, WA, USA). The stimulator PBMCs and other MSCs were inactivated by treatment with 10 µg/mL mitomycin-C (Sigma) for 1 h at 37 °C. To assess suppression of T-cell proliferation, clustering of T-cells was assessed in bright field and BrdU ELISA (Roche, Mannheim, Germany) at 3 days' cultivation. Sample absorbance was measured at 370~492 nm.

Figure S3. Immunosuppression by MSCs from BM, AT, and UCB. Human responder PBMCs were cultured with inactivated allogeneic PBMCs (positive control; MLR). All MSCs significantly reduced the proliferation of alloreactive T-lymphocytes in comparison to MLR. The difference between MSCs was not statistically significant. Error bars represent the means ± SD, $n = 5$; ** $p < 0.01$ *vs*. MLR.

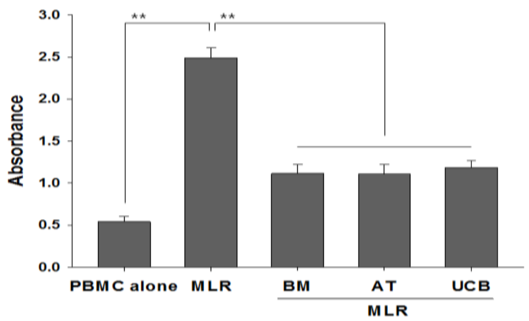

© 2013 by the authors; licensee MDPI, Basel, Switzerland. This article is an open access article distributed under the terms and conditions of the Creative Commons Attribution license (http://creativecommons.org/licenses/by/3.0/).
